# Diagnosis and treatment of nonadjacent cryptococcal infections at the L1 and S1 vertebrae

**DOI:** 10.1007/s00132-016-3349-3

**Published:** 2016-11-17

**Authors:** Qi Lai, Yuan Liu, Xionglong Yu, Xin Lv, Qiang Wang, Yibiao Zhou, Runsheng Guo, Bin Zhang

**Affiliations:** 1 Department of Orthopedics, Artificial Joints Engineering and Technology Research Center of Jiangxi Province, The First Affiliated Hospital of Nanchang University, 330006 Nangchang, Jiangxi China; 2 Multidisciplinary Therapy Center of Musculoskeletal Tumor, The First Affiliated Hospital of Nanchang University, 330006 Nangchang, Jiangxi China

**Keywords:** Cryptococcosis, Bacterial infections and mycoses, Bone diseases, Cancer, Spine, Kryptokokkose, Bakterielle Infektionen und Mykosen, Knochenerkrankungen, Krebs, Wirbelsäule

## Abstract

Cryptococcal spine infections are rare infections that are easy to misdiagnose and difficult to cure. Therefore, we report the case of a 25-year-old man who presented with nonspecific spinal lesions at L1 and S1. The patient underwent surgical removal of the lesions, and specimens were submitted for microbial identification, which identified a cryptococcal infection that was susceptible to amphotericin B. The patient exhibited marked improvement after receiving intravenous amphotericin B and remained asymptomatic (no back pain, fever, or other symptoms) at the 3‑ and 9‑month follow-ups. Similar cases of cryptococcal spine infections are rare, and we believe that our diagnostic findings and treatment experience may help improve the management of this rare disease.

## Zusammenfassung

Kryptokokkeninfektionen an der Wirbelsäule sind selten. Sie sind nicht einfach zu diagnostizieren und schwierig zu behandeln. Aus diesem Grund berichten die Autoren über den Fall eines 25-jährigen Mannes, der sich mit unspezifischen Wirbelläsionen im Bereich L1 und S1 vorstellte. Der Patient unterzog sich einer chirurgischen Entfernung der Läsionen. Es erfolgte eine mikrobiologische Untersuchung von Proben, bei welcher eine Kryptokokkeninfektion identifiziert wurde, die auf Amphotericin B anspricht. Der Zustand des Patienten verbesserte sich deutlich nach der intravenösen Gabe von Amphotericin B, und es traten bei den Nachsorgeuntersuchungen nach 3 und 9 Monaten keine weiteren Symptome auf (kein Rückenschmerz, Fieber oder andere Symptome). Ähnliche Fälle von Kryptokokkeninfektionen an der Wirbelsäule sind selten. Die Autoren sind der Meinung, dass ihre Erfahrung mit den diagnostischen Ergebnissen und der Therapie dabei helfen kann, das Krankheitsmanagement dieser seltenen Erkrankung zu verbessern.

Bone involvement is common in cases of tuberculosis and postoperative infections, and there are a few reports of spinal infections, which were mainly caused by *Mycobacterium tuberculosis* or *Staphylococcus aureus.* Furthermore, fungal spine infections (e.g., Cryptococcosis) are extremely rare. Moreover, cryptococcal infections exhibit symptoms and imaging findings that are extremely similar to tuberculous infections, bacterial infections, and spinal tumors. Thus, fungal infections of the spine are difficult to diagnose, which can delay treatment and ultimately create significant economic, mental, and physical burden for the patient. In the present report, we describe our diagnostic and treatment experiences in an extremely rare case of a 25-year-old man with cryptococcal infection of the L1 and S1 vertebrae, which may help other clinicians manage similar cases of cryptococcal spine infections.

## Case report

The patient provided informed consent for the publication of this report, and we received ethical approval from the ethical review committee of the First Affiliated Hospital of Nanchang University Medical School.

A 25-year-old man presented with a 1-month history of spinal osteosarcoma that was misdiagnosised at a local county hospital, as well as a 5-week history of progressive lower back pain and occasional pain radiating to the left lower limb. The patient had no other medical history. A general physical examination and central nervous system examination upon patient admission revealed normal findings. His erythrocyte sedimentation rate (ESR) was 14 mm/h (normal: 0–20 mm/h) and his C‑reactive protein (CRP) levels were 11.2 mg/l (normal: 0–5.0 mg/l); all other blood test findings were normal.

**Fig. 1:**
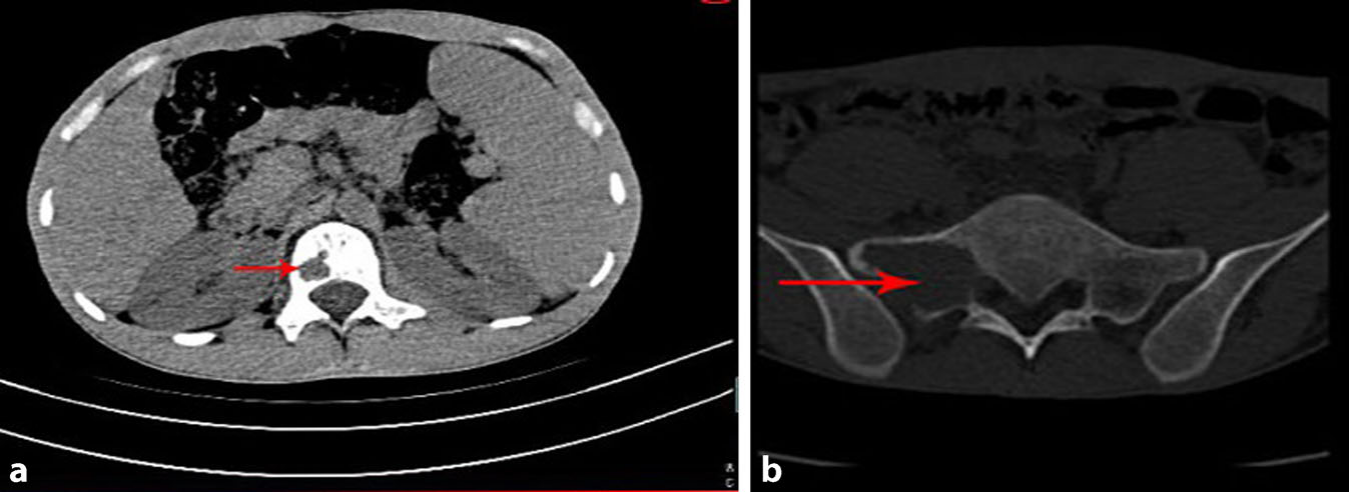
Preoperative computed tomography of S1 and L1 reveals bone destruction and vertebral abnormalities. **a** L1 Vertebral lesions and vertebral invasion. **b** S1 Vertebral lesions and vertebral invasion. *Red arrows* indicate location and extent of the lesions

**Fig. 2:**
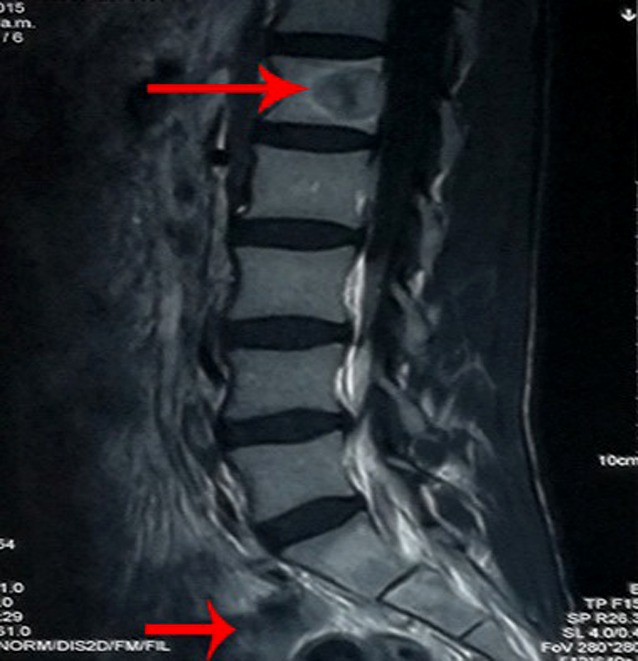
Preoperative magnetic resonance imaging of L1 and S1–S2 reveals bone destruction and vertebral abnormalities. *Red arrows* indicate L1 (*upper arrow*) and S1-S2 (*lower arrow*) lesions in MRI

**Fig. 3:**
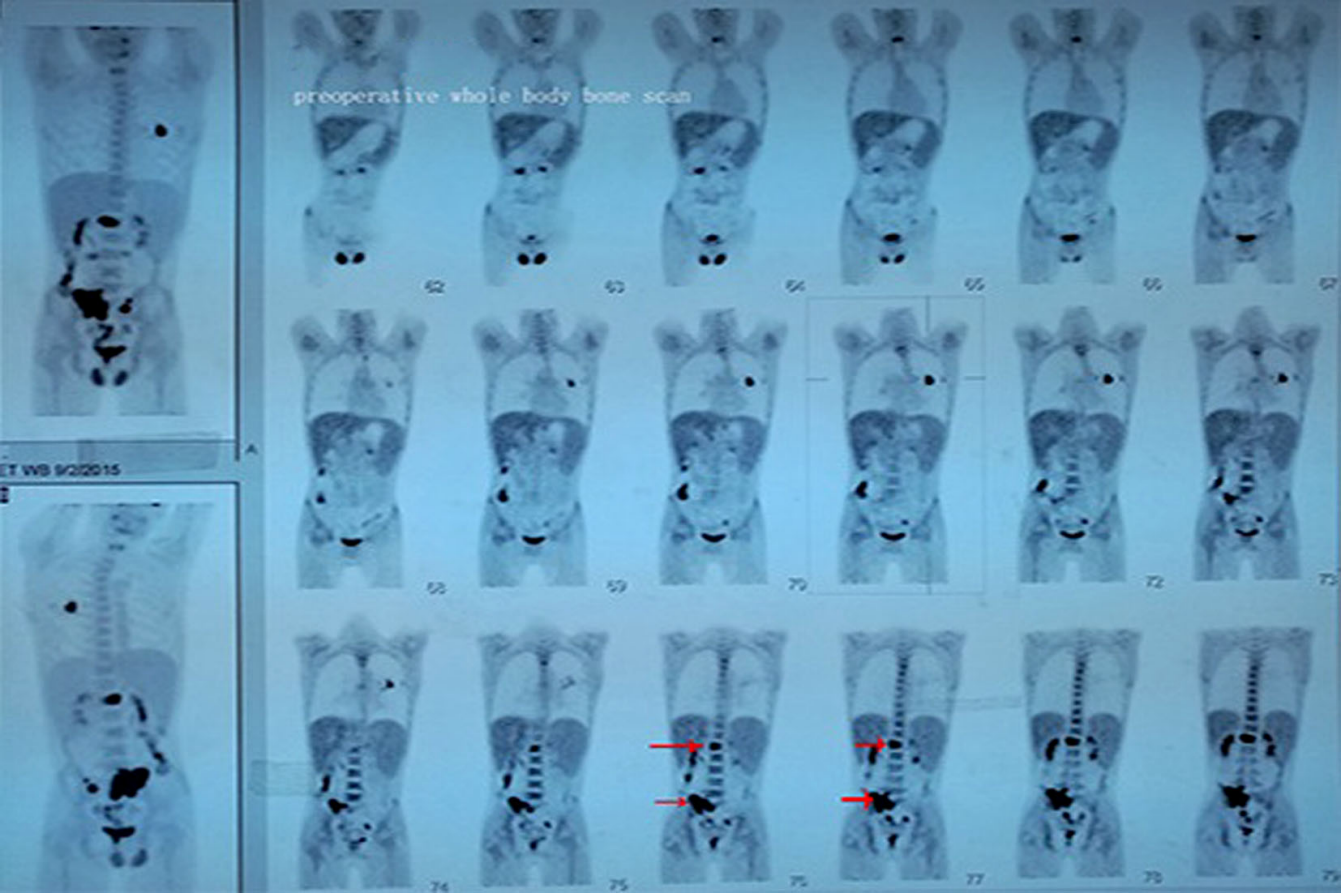
A preoperative whole-body bone scan reveals abnormal bone densities at L1 and S1–S2, which indicated metastatic tumors. *Red arrows* show extent of invasion of lesions in whole-body bone scan

Transverse computed tomography (CT) revealed nonadjacent low-intensity lesions with clear boundaries at L1 and S1 (Fig. 1). Lumbar vertebra magnetic resonance imaging (MRI; Fig. 2) also revealed that the left half of the L1 vertebral body had been destroyed and that the S1 lesion extended into the soft tissues. A whole-body bone scan (Fig. 3) revealed generally clear results, although the L1 and S1–S2 vertebrae exhibited abnormal bone densities, which indicated the possibility of bone tumor. All of these findings indicated a malignant tumor, but the type of tumor could not be diagnosed clearly and needed further examination.

**Fig. 4:**
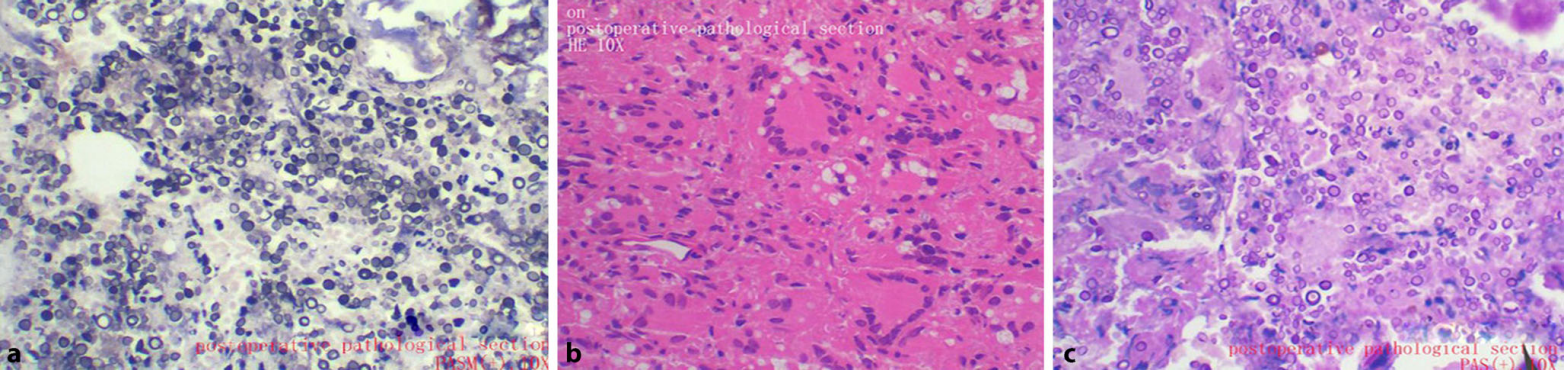
Postoperative pathology reveals epithelioid cells and multinucleated giant cell granuloma formation, with significant caseous necrosis, granuloma, and a diffuse oval body with refraction around the crassicarpa membrane. Positive periodic acid-Schiff (PAS) and periodic acid-silver methenamine (PASM) results indicate a fungal infection. **a** PASM is positive. **b** pathological results: fungal infection. **c** positive periodic acid-schiff (PAS(+))

**Fig. 5:**
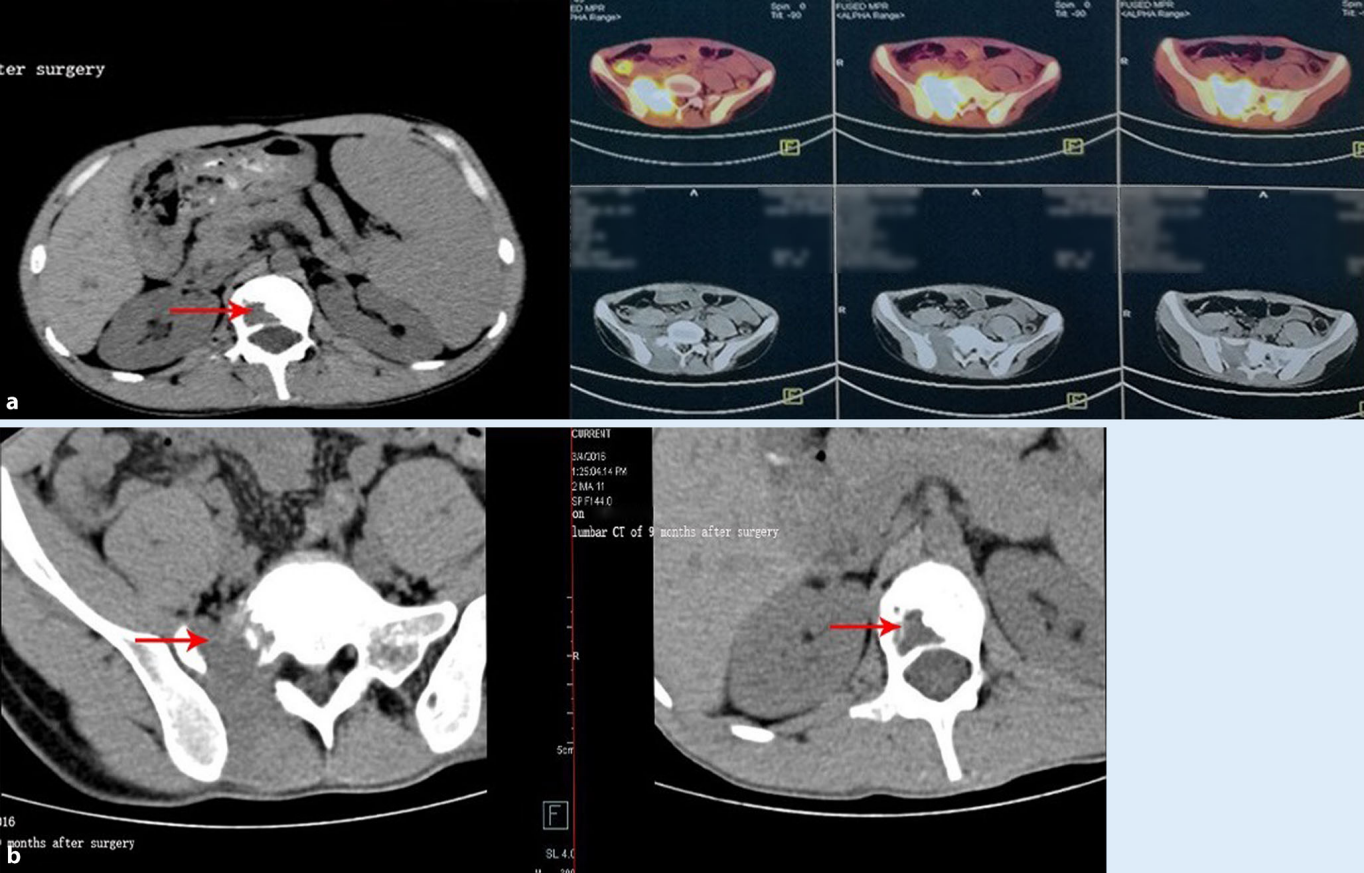
Computed tomography at **a** 3 months and **b** 9 months reveals no symptoms of recurrence and no changes in the lesions. *Red arrows* show improvement of lesions at 3‑month and 9‑month follow-up

Three days after the admission, the patient experienced night-time fever, and blood tests revealed a white blood cell count of 10.2 × 10^9^/l, an ESR of 46 mm/h, and CRP levels of 27.3 mg/l. Based on these findings, the patient agreed to undergo an S1 vertebra puncture biopsy. The biopsy results revealed an infected lesion with massive neutrophil infiltration, and we started treating the patient using intravenous moxifloxacin and teicoplanin. However, the patient continued to exhibit pain radiating to the left lower limb, a fever of up to 39.8 °C, an ESR of 62 mm/h, and CRP levels of 44 mg/l. Therefore, we performed lumbosacral debridement under general anesthesia, and obtained intra-operative purulent tissue specimens for pathological examination and microbial culture. The operative finding was that the S1 to the sacroiliac joint contained a 5 × 6‑cm lesion with a large amount of pus. Thus, the surgeons used a spatula to reduce the lesion, along with physiological saline and iodine, and ultimately used a gelatine sponge with vancomycin to fill the cavity. The postoperative pathology report identified inflammatory changes at L1 and S1, which indicated a fungal infection (Fig. 4). The tissue and blood specimens were sent to the Shanghai Huashan Hospital for microbial identification and drug susceptibility testing, which revealed a cryptococcal infection that was sensitive to amphotericin B. Therefore, we treated the patient using 4 weeks of intravenous amphotericin B (80 mg/day) and then 8 weeks of oral amphotericin B (60 mg/day). The patient did not report lower back pain or symptoms of recurrence at the 3‑month follow-up, and his body temperature, blood results, ESR, and CRP levels were normal. The patient also did not exhibit any symptoms of recurrence or abnormal imaging findings at the 9‑month follow-up (Fig. 5).

## Discussion

Spinal infections are common and are mainly observed in cases of tuberculosis [1] or postoperative infection. In contrast, fungal osteomyelitis is a rare spinal infection [2] and is typically caused by *Aspergillus* (38.2 %) or *Candida* (22.9 %) [3]. Furthermore, cryptococcal lumbosacral vertebra infections are extremely rare. The first reported case of a fungal spine infection involved blastomycosis [4], and Eisen et al. [5] reported the first case of cryptococcosis with lung and spine involvement in 1955. Since 1955, some researchers have reported fungal spine infections (typically involving *Aspergillus* or *Candida*), although no reports described cryptococcal infections until a 2013 study by Zhou et al. [6], who examined cryptococcal lumbar infections in patients with rheumatoid arthritis. Furthermore, Wang et al. [7] described the imaging findings of cryptococcal thoracic spine infections. Based on these characteristics, numerous researchers [1–5] believe that cryptococcosis mainly occurs in the central nervous system and lungs of immunocompromised hosts and can involve any body site or structure. Moreover, researchers generally consider cryptococcal spine infections as a rare and opportunistic infectious disease that should be treated using conservative methods.

In the present case, we encountered a 25-year-old generally healthy man with newly diagnosed cancer and back pain, which we ultimately identified as being related to a cryptococcal infection. Therefore, it appears that otherwise healthy individuals may be vulnerable to these infections, despite the consensus opinion that primary fungal infections are limited to patients with immunodeficiency or immunosuppression [8]. Furthermore, Wong et al. [9] reported that patients rarely exhibit fungal spine infections if they do not have immunodeficiency factors or primary infected lesions, and most cases of cryptococcal spine infections among healthy people are likely misdiagnosed as cancer or tuberculous granuloma. Moreover, Lzzati et al. [10] and others believe that approximately 25 % of spinal infections occur in cases with no means of obtaining a bacteriological diagnosis. Similarly, orthopedic surgeons and radiologists can attempt noninvasive radiological examinations to confirm a fungal spine infection, but there have not been any breakthroughs in this field. Sobottke et al. [11] evaluated the diagnostic value of positron emission tomography, although this modality provided minimal value for diagnosing idiopathic fungal spine infections. Therefore, many patients are not diagnosed at a stage that would facilitate conservative treatment and must undergo surgery.

In the present case, we originally considered the possibility of a spinal tumor, and only identified the fungal infection after we performed S1 biopsy and lumbosacral debridement. Thus, an early diagnosis can only be achieved through clinician awareness, a detailed medical history, a careful physical examination, relevant laboratory testing, and imaging findings. We also suggest that, in cases with suspicious symptoms, patients should quickly undergo testing for procalcitonin levels, ESR, CRP levels, and spinal MRI. This is because spinal infections are characterized by an elevated ESR (sensitivity: 76–81 %) [12], elevated CRP levels (sensitivity: 90–93 %) [13], and elevated procalcitonin levels (sensitivity: 95–97 %) [13]. Procalcitonin testing is especially sensitive at the early stage, and MRI is also widely used to evaluate inflammation in the vertebral body and disk, as Modic et al. [14] found that MRI provided a sensitivity of 96 % for identifying vertebral osteomyelitis. Moreover, if the results of these tests indicate a spinal infection, puncture biopsy and culturing should be immediately performed to confirm the diagnosis. If the results suggest a fungal infection, the fungus should be identified and the fungal spores viewed under a microscope [15]. This is because the histological findings of cryptococcal spine infections are nonspecific (e. g., a sequestrum and/or abscess), and visual evaluation of the spores is essential for diagnosing a cryptococcal spine infection. Therefore, biopsy, microbial culture, and fungal spore examination can help identify the fungus, and then anti-fungal therapy should be implemented as soon as possible.

In the present case, the patient underwent surgical removal of the spinal lesions, with postoperative saline irrigation for 1 week and removal of specimens for pathological examination and microbial culture. These tests revealed a fungal infection (Fig. 5), and fungus identification and drug susceptibility experiments ultimately identified a cryptococcal infection that was sensitive to amphotericin B. The patient’s symptoms significantly improved after 4 weeks of intravenous amphotericin B, and we observed obvious decreases in the values for ESR, CRP, and procalcitonin. Therefore, we provided oral amphotericin B for 8 weeks after the patient’s discharge, and at the 3‑month and 9‑month follow-ups no symptoms of recurrence or lesion proliferation were detected.

## Conclusion

We encountered a 25-year-old man with a rare cryptococcal spine infection that presented as newly diagnosed cancer and lower back pain. This case indicates that this infection should be considered in endemic regions, among immunodeficient patients, and also in the normal population. However, the symptoms of cryptococcosis are atypical and difficult to diagnose using a simple physical examination; therefore, biopsy, microbial culture, and fungal spore evaluation are essential steps that are needed to make a definitive diagnosis. Murray et al. [16] recommended 12 weeks of intravenous voriconazole and oral fluconazole for the treatment of fungal infections, although we recommend that cases of spinal cryptococcosis with an early diagnosis should treat using 12 weeks of amphotericin B (4 weeks of intravenous amphotericin B and 8 weeks of oral amphotericin B). Moreover, surgical treatment with postoperative physiological saline irrigation for 7–12 days [17] should be considered in cases without an early diagnosis, with delayed treatment, with severe symptoms, or cases that are not appropriate for conservative treatment.
